# Promising operational stability of high-efficiency organic light-emitting diodes based on thermally activated delayed fluorescence

**DOI:** 10.1038/srep02127

**Published:** 2013-07-03

**Authors:** Hajime Nakanotani, Kensuke Masui, Junichi Nishide, Takumi Shibata, Chihaya Adachi

**Affiliations:** 1 Center for Organic Photonics and Electronics Research (OPERA), Kyushu University, 744 Motooka, Nishi, Fukuoka 819-0395, Japan; 2 Innovative Organic Device Laboratory, Institute of Systems, Information Technologies and Nano-technologies (ISIT), 744 Motooka, Nishi, Fukuoka 819-0395, Japan; 3 Advanced Research Laboratories, Fujifilm Co., 577 Ushijima, Kaisei, Ashigarakami, Kanagawa 258- 8577, Japan; 4 OLED R&D Department, Research and Development Division, Japan Display Inc., Landic 2nd Bdg., 3-7-1, Nishi-Shinbashi, Minato, Tokyo 105-0003, Japan; 5 International Institute for Carbon Neutral Energy Research (WPI-I2CNER), Kyushu University, 744 Motooka, Nishi, Fukuoka 819-0395, Japan; 6 These authors contributed equally to this work.

## Abstract

Organic light-emitting diodes (OLEDs) are attractive for next-generation displays and lighting applications because of their potential for high electroluminescence (EL) efficiency, flexibility and low-cost manufacture. Although phosphorescent emitters containing rare metals such as iridium or platinum produce devices with high EL efficiency, these metals are expensive and their blue emission remains unreliable for practical applications. Recently, a new route to high EL efficiency using materials that emit through thermally activated delayed fluorescence (TADF) was demonstrated. However, it is unclear whether devices that emit through TADF, which originates from the contributions of triplet excitons, are reliable. Here we demonstrate highly efficient, stable OLEDs that emit via TADF by controlling the position of the carrier recombination zone, resulting in projected lifetimes comparable to those of tris(2-phenylpyridinato)iridium(III)-based reference OLEDs. Our results indicate that TADF is intrinsically stable under electrical excitation and optimization of the surrounding materials will enhance device reliability.

Organic light-emitting diodes (OLEDs) have advantages such as high electroluminescence (EL) efficiency, flexibility and low manufacturing costs, and are attracting increasing attention for use in advanced displays and lighting. Usually, a phosphorescent emitter, such as iridium^1^,^2^,^3^ or platinum^4^,^5^ complexes, is used so that both the 25% of singlet excitons and the 75% of triplet excitons can be used for EL. All excitons are accessible in such systems because of the presence of strong spin-orbit coupling, which enhances intersystem crossing (ISC) from the lowest singlet excited state (S_1_) to the lowest triplet excited state (T_1_), and from T_1_ to the ground state (S_0_), resulting in an internal EL quantum efficiency (η_int_) of nearly 100%^6^.

Recently, we produced high-efficiency OLEDs by using a new route to produce thermally activated delayed fluorescence (TADF)^7^,^8^,^9^, achieving an external EL quantum efficiency (η_EQE_) of 20% that was comparable to that of phosphorescent OLEDs^10^. TADF originates from the contributions of triplet excitons, which are accessed through efficient up-conversion from T_1_ to S_1_ by thermal activation. To obtain efficient TADF, a small energy gap between the S_1_ and T_1_ states (ΔE_ST_) is required, which is realized in molecules with a small overlap between their highest occupied molecular orbital (HOMO) and lowest unoccupied molecular orbital (LUMO). Recent designs of molecules that exhibit TADF have been based on a combination of the donor and acceptor units, and have achieved ΔE_ST_ comparable to or lower than the thermal energy of 27 meV at 300 K.

Phthalonitrile derivatives such as (4s,6s)-2,4,5,6-tetra(9H-carbazol-9-yl)isophthalonitrile (4CzIPN) are promising emitters for highly efficient TADF-based OLEDs^10^. However, the 4CzIPN-based OLED, which was described in Ref. 10, showed a rapid decrease in luminance under a constant DC current, resulting in a half-life (LT 50) of less than 10 h. We need to determine if the molecular design based on a donor-acceptor structure is the origin of the short lifetime of this OLED.

In this work, to investigate the possibility of TADF-based OLEDs with high operational stability, we carefully designed a device architecture that included exciton-blocking layers (EBLs) at the interfaces of the emission layer (EML) and optimized the concentration of the emitter. Here we show that expanding the carrier recombination zone to enhance the electron carrier injection efficiency significantly affects the operational stability of the device. We realize lifetimes of more than 2,500 h at an initial luminance of 1,000 cd/m^2^ in TADF-based OLEDs while maintaining a high η_EQE_ that is comparable to those of OLEDs containing iridium complexes.

## Results

### Design of OLED configuration

To maximize the TADF efficiency, we prepared a guest-host system that escapes concentration quenching, similar to conventional fluorescence and phosphorescence based OLEDs. Also, because TADF is based on an up-conversion process of triplet excitons into a singlet state, improper protection of the triplet excitons induces Dexter energy transfer into the surrounding molecules during the lifetime of the guest's triplet state, which resulted in exciton quenching. It is therefore necessary to introduce host and carrier transport molecules with higher triplet energies than that of a guest TADF molecule.

**Figure 1:**
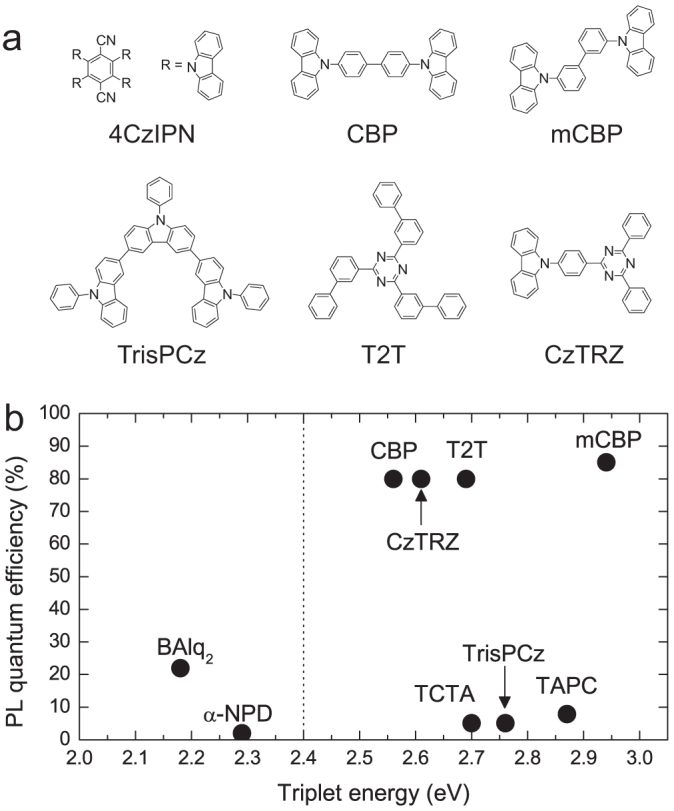
Molecular structures and optical characterization of 4CzIPN co-deposited films with various hosts. (a) Molecular structures used in this study. (b) PL quantum efficiencies of 4CzIPN co-deposited films with various hosts as a function of host triplet energy. The dashed line indicates the triplet energy level of 4CzIPN. The triplet energies of the host materials were estimated from the peak emission wavelengths of phosphorescence spectra of the films measured at low temperature (5 K).

The high photoluminescence (PL) quantum efficiency (Φ_PL_) of an EML is one of the main factors in obtaining a high η_int_ in OLEDs. The use of 3,3-di(9H-carbazol-9-yl)biphenyl (mCBP) as a host matrix with 6 wt.%-4CzIPN showed a slightly higher Φ_PL_ = 85 ± 2% than the Φ_PL_ = 80 ± 2% of 4,4′-bis(carbazol-9-yl)biphenyl (CBP), as shown in Fig. 1(b), indicating that mCBP with T_1_ = 2.90 eV^11^ traps the triplet excitons of 4CzIPN (T_1_ = 2.4 eV) well, and does so better than CBP with T_1_ = 2.55 eV. Also, in a dilute concentration of 1 wt.%, the maximum Φ_PL_ = 94 ± 2% was obtained, as shown in Fig. S1(a). We therefore adopted mCBP as the host matrix.

To confirm the confinement behavior of the triplet excitons of 4CzIPN at the interface between a 4CzIPN doped EML and either a hole transport layer (HTL) or an EBL, Φ_PL_ values of co-deposited films of 4CzIPN in various host matrices were evaluated (Fig. 1(b)). We found that the hole transport materials, such as N,N′-bis(naphthalen-1-yl)-N,N′-bis(phenyl)-benzidine (α-NPD, where T_1_ = 2.3 eV, HOMO = 5.5 eV), tris(4-carbazoyl-9-ylphenyl)amine (TCTA, where T_1_ = 2.76 eV^12^, HOMO = −5.9 eV), 1,1-bis[(di-4-tolylamino)phenyl]cyclohexane (TAPC, where T_1_ = 2.87 eV^13^, HOMO = −5.5 eV) and 9,9′,9″-triphenyl-9H,9′H,9″H-3,3′:6′,3″-tercarbazole (Tris-PCz, where T_1_ = 2.7 eV, HOMO = −5.6 eV) completely quench the excitons of 4CzIPN when they are used as hosts, giving very low Φ_PL_ values of 2%, 5%, 7% and 5%, respectively. This is because of the low triplet energy level of α-NPD, the strong electron donation ability of the triphenylamine units, and the rather shallow HOMO levels of these materials (Fig. S1(c)). Therefore, a lack of exciton confinement occurs at the interfaces between the HTLs and EMLs. However, we observed no clear reduction in η_EQE_ in the OLEDs, suggesting that the carrier recombination zone is away from the interface between the HTLs and EMLs.

We also determined Φ_PL_ for co-deposited films of 6 wt.%-4CzIPN and various EBLs, including bis(2-methyl-8-quinolinolate)-4-(phenylphenolato)aluminum (BAlq_2_)^14^, 9-[4-(4,6-diphenyl-1,3,5-triazin-2-yl)phenyl]-9H-carbazole (CzTRZ) and 2,4,6-tris(biphenyl-3-yl)-1,3,5-triazine (T2T)^15^ (Fig. 1(b)). A low Φ_PL_ of 22% was obtained in the BAlq_2_ host, while a high Φ_PL_ of 80% was obtained in the CzTRZ and T2T host matrices. This clearly indicates that the BAlq_2_ host quenches a triplet exciton of 4CzIPN because of the low triplet energy level of BAlq_2_.

**Figure 2:**
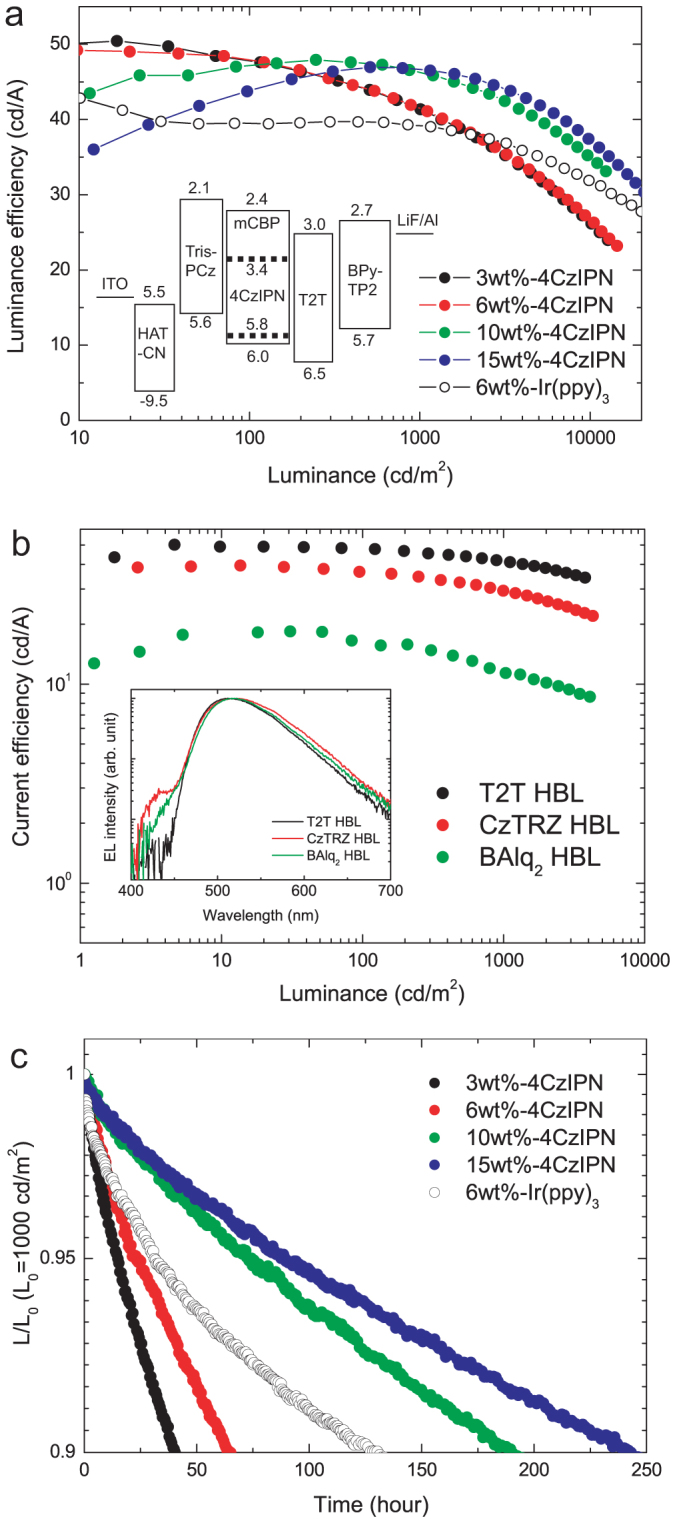
Performance of OLEDs with TADF emitters. (a) Luminance efficiency (cd/A) vs. luminance (cd/m^2^) characteristics for 3, 6, 10 and 15 wt.% 4CzIPN-doped OLEDs. Inset: Energy diagram of the fabricated devices. HOMO levels were measured by photoelectron spectroscopy (Riken Keiki, AC-3). *E*_g_ values were estimated from the absorption edges of the films. (b) Current efficiency (cd/A) vs luminance (cd/m^2^) in 6 wt.%-4CzIPN doped OLEDs with various HBL materials. Inset: EL spectra of the 6 wt.%-4CzIPN doped OLEDs with various HBL materials at luminance of 1,000 cd/m^2^. (c) Normalized luminance of a 4CzIPN emitter-based OLED as a function of operating time at initial luminance of approximately 1,000 cd/m^2^.

Based on this preliminary PL experiment, OLEDs were fabricated by the sequential deposition of a 10 nm-thick layer of dipyrazino[2,3-f:20,30-h]quinoxaline-2,3,6,7,10,11-hexacarbonitrile (HAT-CN) as a hole injection layer (HIL), a 30 nm-thick layer of Tris-PCz as an HTL, an EML consisting of a 30 nm-thick layer of mCBP doped with various concentrations of 4CzIPN, a 10 nm-thick layer of T2T as an EBL, a 40 nm-thick layer of 2,7-bis(2,20- bipyridine-5-yl)triphenylene (BPy-TP2)^16^ as an ETL, a 0.8 nm-thick lithium fluoride (LiF) electron injection layer (EIL), and a 100 nm-thick aluminum (Al) cathode. An energy level diagram of the devices used in this study is shown in the inset of Fig. 2(a). We also fabricated a fac-tris(2-phenylpyridinato)iridium(III) (Ir(ppy)_3_)-based OLED to act as a reference device.

### Electrical characteristics

The current efficiency (cd/A) vs. current density (*J*) characteristics of the 3, 6, 10 and 15 wt.% 4CzIPN-doped devices are shown in Fig. 2(a). These devices exhibited high luminance efficiencies of 50 cd/A (η_EQE_ = 17 ± 0.5%), 49 cd/A (η_EQE_ = 15.6 ± 0.5%), 48 cd/A (η_EQE_ = 14.2 ± 0.5%) and 47 cd/A (η_EQE_ = 14.0 ± 0.5%) at J = 0.01, 0.04, 0.5 and 1.0 mA/cm^2^, respectively. For the 3 and 6 wt.% 4CzIPN-doped devices, the highest η_EQE_ was observed at low current density (<0.1 mA/cm^2^). In contrast, the 10 and 15 wt.% 4CzIPN-doped devices showed a gradual increase in η_EQE_ with increasing *J*, indicating that the balance of the hole and electron carrier injection changes with the dopant concentration. At rather high current densities, a high η_EQE_ of 13.8 ± 0.5% (46 cd/A) was obtained at 1,000 cd/m^2^ for the 10 wt.%-doped device, which is higher than those of the 3 and 6 wt.%-doped devices.

We derived the theoretical maximum η_EQE_ for the 10 wt.%-doped OLED using the following equation^8^: 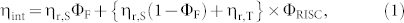
where η_r,S_ is the proportion of singlet excitons (25%) and η_r,T_ is the proportion of triplet excitons (75%) produced under electrical excitation. From transient PL analysis (Fig. S2), the contributions from the prompt component (Φ_F_) and the TADF component (Φ_TADF_) were estimated to be 27% and 51%, respectively, resulting in a reverse ISC (RISC) efficiency (Φ_RISC_) of 69%, which was estimated from the relation (1 − Φ_F_) Φ_RISC_ = Φ_TADF_. The theoretical maximum η_EQE_ is thus estimated to be 14.2%, assuming a light out-coupling efficiency of 20%. Therefore, near-complete carrier recombination and exciton confinement in the EML were realized in the 10 wt.%-doped device.

Then, we changed the hole blocking layer (HBL) from T2T to CzTRZ and BAlq_2_ to examine the effect of the recombination zone position on the device performance. The OLEDs with CzTRZ and BAlq_2_ HBLs had inferior characteristics when compared with those of the devices with T2T HBLs (Fig. 2(b)). In particular, an emission originating from CzTRZ was observed (Fig. 2(b), inset), indicating both the inefficient carrier confinement at the EML/EBL interface because of the shallow HOMO of the CzTRZ layer (−6.1 eV) and the location of the carrier recombination zone near the EML/HBL interface.

### Device operational stability

Figure 2(c) shows the normalized luminance of the 4CzIPN-based OLEDs as a function of operational time at an initial luminance L_0_ of 1,000 cd/m^2^ and Table 1 summarizes the OLED properties. We observed a significant dependence of the operational lifetime, defined as operation at 90% of the initial luminance (LT90), on the 4CzIPN doping concentration. At low doping concentrations, LT90 was only 40 and 65 h for the 3 and 6 wt.%-doped devices, respectively. In contrast, LT90 values of 190 and 253 h were observed for the 10 and 15 wt.%-doped devices, respectively. These results show that the 4CzIPN concentration strongly affects the operational stability of these devices.

To predict LT50 for the devices containing 10 and 15 wt.% 4CzIPN at L_0_ = 1,000 cd/m^2^, we estimated an acceleration factor of 1.92 for each device from the lifetime measurements at L_0_ = 2,000, 5,000 and 10,000 cd/m^2^ using the following well-known equation^17^: 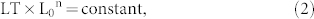
where LT is the operational lifetime and n is an acceleration factor. Based on Eq. (2), LT50 is predicted to be 1,900 h for the 10 wt.%-doped device and 2,800 h for the 15 wt.%-doped device, which is comparable to that predicted for a 6 wt.% Ir(ppy)_3_-doped device (4,500 h), as summarized in Table 1.

## Discussion

**Figure 3:**
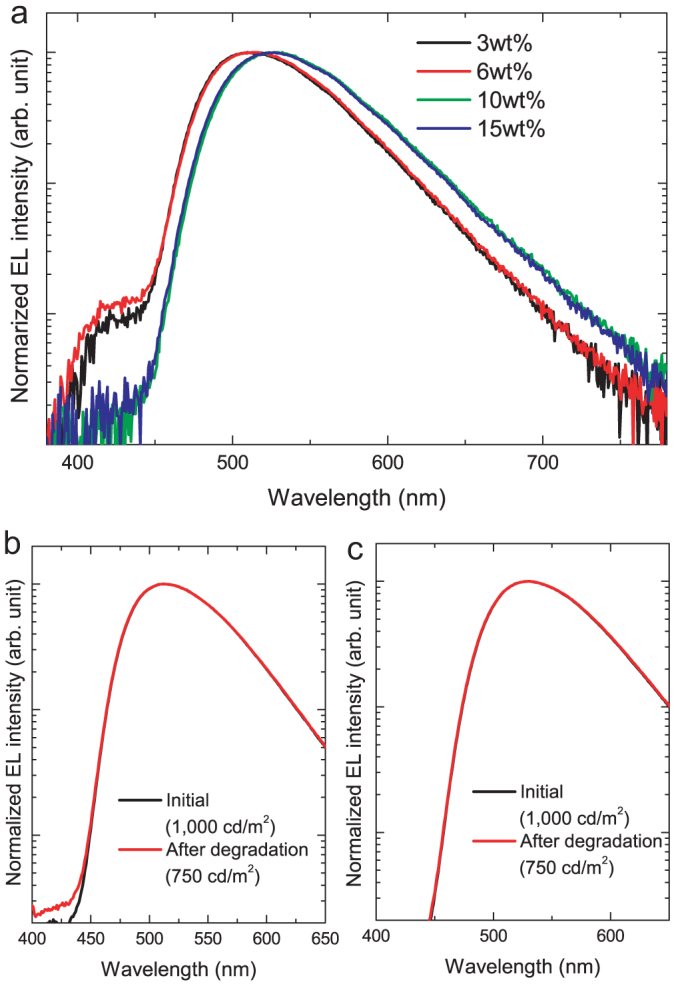
EL spectra of OLEDs containing TADF emitters. (a) EL spectra of 3, 6, and 10 wt.% 4CzIPN-doped OLEDs at luminance of 20,000 cd/m^2^. (b) EL spectra of 3 wt.% 4CzIPN-doped OLEDs before and after device degradation. (c) EL spectra of 15 wt.% 4CzIPN-doped OLEDs before and after device degradation.

There are several possible reasons for the enhancement of LT50 with an increase in the doping concentration. We envisage that the position of the carrier recombination zone strongly affects the operational stability of these devices. In fact, although the EL from only S_1_ of 4CzIPN was observed at 1,000 cd/m^2^ in all devices, another emission peak around the deep blue region was observed in the 3 and 6 wt.%-doped devices at 20,000 cd/m^2^, suggesting that the carrier recombination zone position changes in these devices, as shown in Fig. 3(a). Also, after the device begins to degrade (LT75 = 164 h), an additional emission signal was observed from the 3 wt.%-doped devices (Fig. 3(b)). This signal was similar to that observed from the pristine device at 20,000 cd/m^2^, indicating that the carrier recombination zone moves during constant operation. However, no additional emission signal was observed from the 15 wt.%-doped devices (Fig. 3(c)), even after the device began to degrade (LT75 = 820 h). These results indicate the absence of the carrier recombination zone at the EML/EBL interface in the 15 wt.%-doped devices.

**Figure 4:**
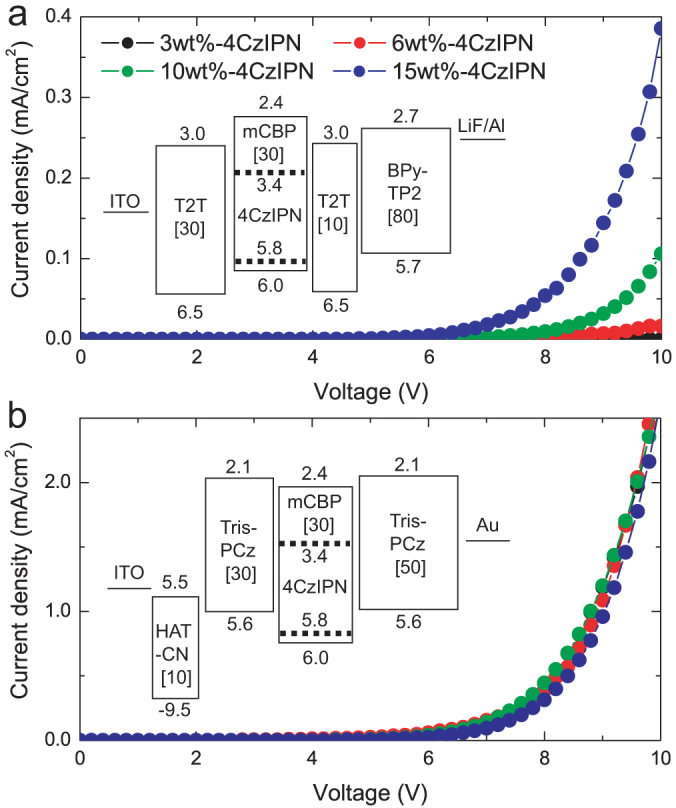
*J-V* characteristics of single carrier transport devices. (a) *J* vs. *V* characteristics for 3, 6, 10 and 15 wt.% 4CzIPN-doped EODs. Inset: Energy diagram of the EODs. (b) *J* vs. *V* characteristics for 3, 6, 10 and 15 wt.% 4CzIPN-doped HODs. Inset: Energy diagram of the HODs.

To determine the effect of the doping concentration on the carrier transport properties, electron-only devices (EODs) and hole-only devices (HODs) were fabricated, as shown in the insets of Fig. 4(a) and (b), respectively. Although the 3 wt.%-doped EOD showed a very low J of 10^−3^ mA/cm^2^ at 10 V, the driving voltage decreased significantly as the 4CzIPN doping concentration was increased, resulting in J values of 0.1 and 0.4 mA/cm^2^ at 10 V for the 10 and 15 wt.%-doped EODs, respectively (Fig. 4(a)). In contrast, the HODs showed almost no dependence on the driving voltage as the 4CzIPN doping concentration increased, as shown in Fig. 4(b). This clearly indicates that only the electron injection/transport efficiency is enhanced by an increase in the doping concentration. Therefore, an increase in the doping concentration enhances the efficiency of electron injection from T2T into the EML and subsequent transport in the EML. These results also suggest that the carrier recombination zone shifts from the EML/ETL interface into the bulk of the EML when the doping concentration is as high as 10 wt.%. The LUMO level of 4CzIPN (−3.4 eV) is located considerably lower that of mCBP (−2.4 eV), while their HOMO levels are similar (−5.8 and −6.0 eV for 4CzIPN and mCBP, respectively). Therefore, the 4CzIPN molecules in an mCBP host act as strong electron trapping sites, so the recombination process mainly involves direct carrier injection, transport and recombination at the 4CzIPN molecules. A higher dopant concentration expands the exciton formation sites into the bulk of the EML, which produces highly reliable OLEDs^18^,^19^. In addition, reduction of the hole accumulation at the interface between the EML and the EBL is another possible reason for the enhancement of the device operational stability^20^. Also, because the undesired degradation products of carbazole derivatives have been identified after device degradation^21^, the reduction in the excited state formation on the mCBP host molecules by enhancement of the direct electron injection and transport from a T2T layer to 4CzIPN, e.g., by direct exciton formation at 4CzIPN, is another possible reason for the enhanced device stability.

Finally, we consider the possibility of further enhancement of the device reliability. In this study, we adopted conventional host and carrier transport materials and obtained comparable degradation lifetimes for 4CzIPN and Ir(ppy)_3_. This is an encouraging sign that the emitter itself is quite stable for redox and oxidation processes under electrical excitation. Thus, because Ir(ppy)_3_ derivative-based OLEDs with optimum materials and device architectures have realized very long lifetimes of over 100,000 hrs.^22^,^23^, we can expect further improvements in the device reliability when TADF is combined with the best possible combination of surrounding materials and device architectures.

In summary, we clarified that the operational lifetime of 4CzIPN-based OLEDs depends strongly on the emitter concentration in the EML and demonstrated highly efficient TADF-based OLEDs (η_EQE_ of 13.9 ± 0.5% at 1,000 cd/m^2^) with excellent operational stability, showing LT50 of 2,800 h at 1,000 cd/m^2^ and of over 10,000 h at 500 cd/m^2^. We also found that the 4CzIPN molecules act as strong electron trapping sites in the mCBP EML, and that the position of the recombination zone strongly affects the operational lifetime of these devices. Our results confirm that TADF-based OLEDs show great potential for realization of both high efficiency and operational stability.

## Methods

### Sample preparation and characterization for photoluminescence

Samples for the optical measurements were fabricated by co-depositing host materials and 6 wt.% 4CzIPN with a thickness of 50 nm on a quartz substrate. The PL quantum efficiency (Φ_PL_) was measured by an absolute PL quantum yield measurement system (C11347-01, Hamamatsu Photonics, Japan) under a nitrogen gas flow with excitation wavelengths of 337 or 275 nm. The low-temperature PL intensity and the emission lifetime were measured using a streak camera (C4334, Hamamatsu Photonics, Japan) and a cryostat (Iwatani Industrial Gases Co., Japan) with a nitrogen gas laser (MNL200, Laser Technik, Germany) as the excitation light source under a pressure of about 3 Pa.

### Sample preparation and characterization for electroluminescence

Green TADF-based OLEDs with an effective area of 1 mm^2^ were fabricated on 110 nm-thick indium tin oxide (ITO)-coated glass substrates with a 2 mm stripe pattern. Deposition was performed under vacuum at pressures from 5 × 10^−4^ to 5 × 10^−5^ Pa. After fabrication, the devices were immediately encapsulated with glass lids using epoxy glue in nitrogen-filled glove boxes (O_2_ < 0.1 ppm, H_2_O < 0.1 ppm). A commercial calcium oxide desiccant (Dynic Co., Japan) was included in the encapsulated package. The devices were exposed once to nitrogen gas after the formation of the organic layers because a metal mask was included to define the cathode area. The J-V-luminance characteristics were evaluated using a Keithley 2400 source meter and an absolute η_EQE_ measurement system (C9920-12, Hamamatsu Photonics, Japan). The operational lifetime was measured using a luminance meter (CS-2000, Konica Minolta, Japan) at a constant DC current at room temperature.

## Author Contributions

The experiments were conceived and designed by H.N. and K.M., and were carried out by H.N., K.M. and J.N. H.N. and C.A. wrote the manuscript. The project was supervised by T.S. and C.A. All the authors discussed the results and contributed to the article.

## Supplementary Material

Supplementary Information Supplementary Info File #1

## Figures and Tables

**Table 1:** Comparison of characteristics of OLEDs based on different dopant concentrations. The maximum values were obtained at *J* = 0.01, 0.04, 0.5, 1.0 and 0.02 mA/cm^2^ in 3, 6, 10 and 15 wt.% 4CzIPN-doped devices and a 6 wt.% Ir(ppy)_3_-doped device, respectively

EML	Current efficiency (cd/A) (@1,000 cd/m^2^)	Power efficiency (lm/W) (@1,000 cd/m^2^)	η_EQE_ (%) (@1,000 cd/m^2^)	LT90 (hour)	LT50 (hour)
3wt%-4CzIPN	50.0 (41.4)	35.7 (20.9)	17.0 (13.4)	40	506
6wt%-4CzIPN	49.2 (41.1)	33.5 (19.6)	15.6 (13.1)	65	685
10wt%-4CzIPN	47.9 (46.6)	32.7 (28.1)	14.2 (13.8)	190	ca. 1,900
15wt%-4CzIPN	47.0 (46.5)	30.7 (28.1)	14.0 (13.9)	243	ca. 2,800
6wt%-Ir(ppy)_3_	42.9 (39.1)	32.1 (19.2)	11.8 (11.1)	130	ca. 4,500
