# Supplementary material for: Promising operational stability of high-efficiency organic light-emitting diodes based on thermally activated delayed fluorescence
Source: Sci Rep. 2013 Jul 3;3:2127. doi: 10.1038/srep02127 (PMC3705585; doi:10.1038/srep02127)
Supplement: Supplementary Information — Supplementary Info File #1 [file srep02127-s1.pdf]

Supplementary Information

Promising operational stability of high-efficiency organic light-emitting diodes based on thermally activated delayed fluorescence

Hajime Nakanotani[1,2], Kensuke Masui[1,3], Junichi Nishide[1], Takumi Shibata[1,4] & Chihaya Adachi[1,2,5]

[1]Center for Organic Photonics and Electronics Research (OPERA), Kyushu University, 744 Motooka, Nishi, Fukuoka 819-0395, Japan

[2]Innovative Organic Device Laboratory, Institute of Systems, Information Technologies and Nano-technologies (ISIT), 744 Motooka, Nishi, Fukuoka 819-0395, Japan

[3]Advanced Research Laboratories, Fujifilm Co., 577 Ushijima, Kaisei, Ashigarakami, Kanagawa 258- 8577, Japan

[4] OLED R&D Department, Research and Development Division, Japan Display Inc. Landic 2nd Bdg., 3-7-1, Nishi-Shinbashi, Minato, Tokyo 105-0003, Japan.

[5]International Institute for Carbon Neutral Energy Research (WPI-I2CNER), Kyushu University, 744 Motooka, Nishi, Fukuoka 819-0395, Japan

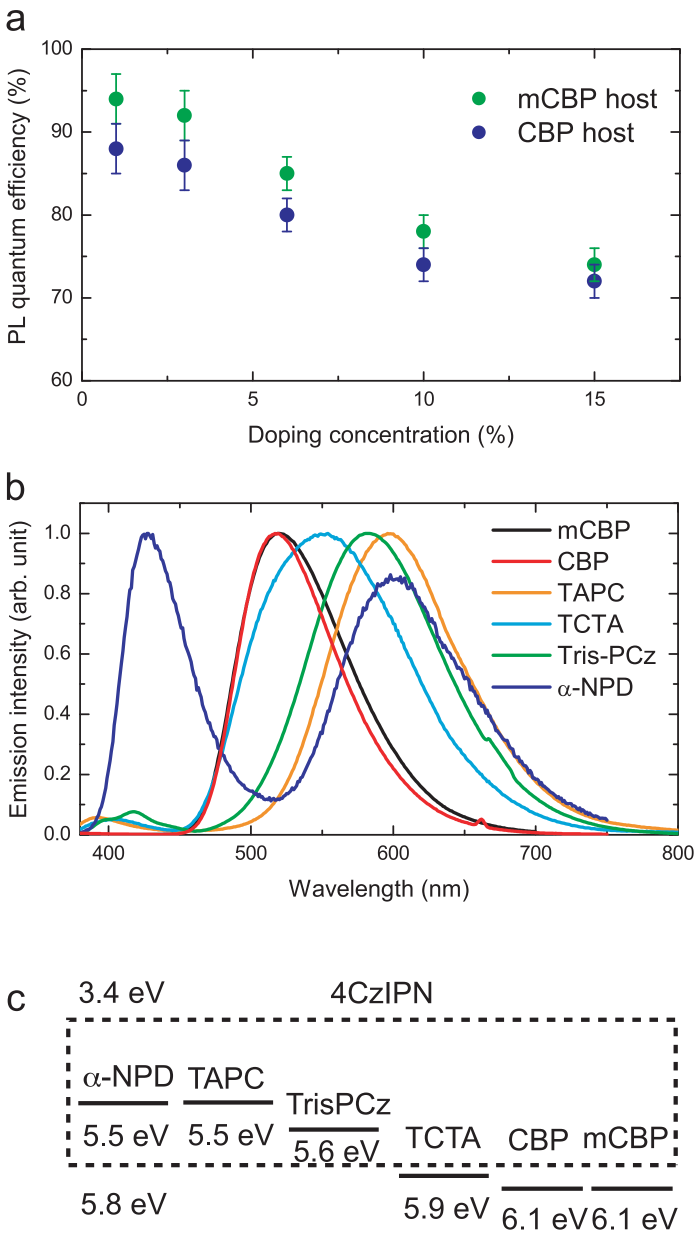

Supplementary Figure S1. | Optical characterization of 4CzIPN co-deposited films with various hosts. Dependence of photoluminescence quantum efficiency (Φ_{PL}) of 4CzIPN co-deposited thin films with CBP and mCBP hosts on 4CzIPN concentration. b) PL spectrum in 4CzIPN co-deposited thin films with various hole transport materials. c) Energy diagram of the HTL and 4CzIPN.

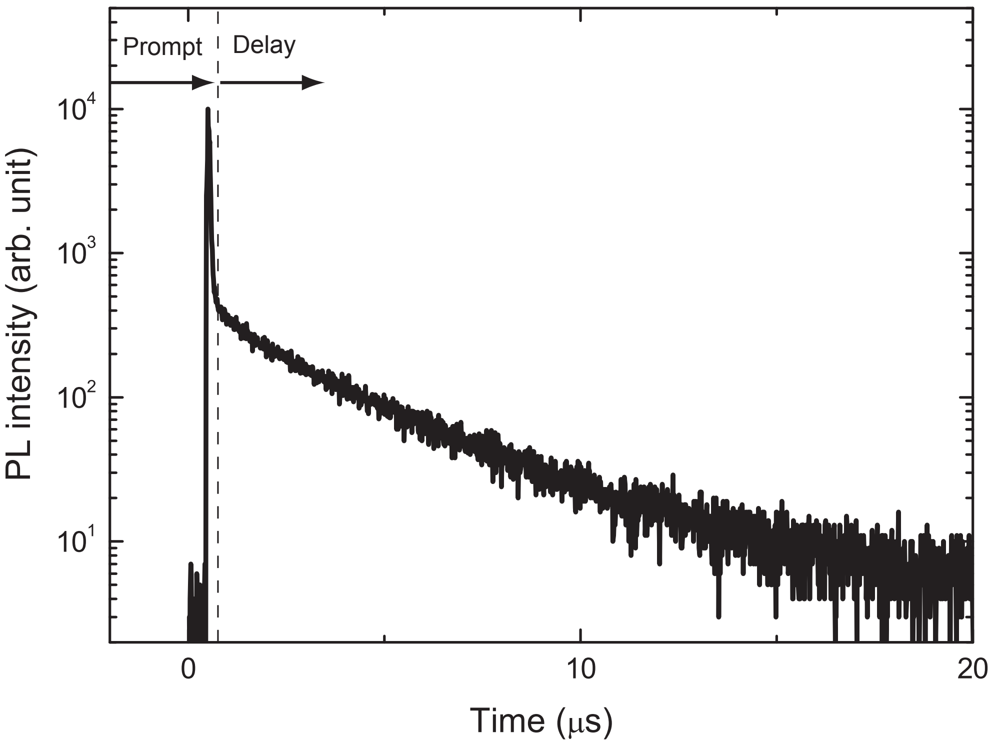

Supplementary Figure S2. | Transient decay curve of 10 wt%-4CzIPN:mCBP co-deposited film. A transient PL for the 6 wt%-4CzIPN : mCBP co-deposited film at room temperatures.
